# Impact of Tai Chi Therapy on Fatigue and Cognitive Function in Individuals With Chronic Fatigue Syndrome: Protocol for a Pilot Randomized Controlled Trial

**DOI:** 10.2196/65958

**Published:** 2025-04-25

**Authors:** Bin Wang, Xiaodong Zhang, Ping Lu, Pingping Sun, Tianxiang He

**Affiliations:** 1 Department of Physical Education Shanghai University of Traditional Chinese Medicine Shanghai China; 2 Department of Tuina Yueyang Hospital of Integrated Traditional Chinese and Western Medicine Shanghai University of Traditional Chinese Medicine Shanghai China; 3 College of Acupuncture and Massage Shanghai University of Traditional Chinese Medicine Shanghai China; 4 College of Rehabilitation Science Shanghai University of Traditional Chinese Medicine Shanghai China; 5 Department of Tuina Shuguang Hospital Affiliated to Shanghai University of Traditional Chinese Medicine Shanghai China

**Keywords:** Tai Chi, chronic fatigue syndrome, cognitive impairment, ANT, N-Back, fatigue, cognitive function, cognitive, protocol, protocols, randomized controlled trial, RCT, controlled trial, controlled trials, psychosomatic disorder, cognition, therapy, efficacy, safety, CFS, intervention, data analysis

## Abstract

**Background:**

Chronic fatigue syndrome (CFS) is a psychosomatic disorder characterized by persistent fatigue, primarily involving physical and mental exhaustion, with greater emphasis on the latter. This leads to a deterioration in concentration and memory. These symptoms affect cognitive functions, including attention and memory, to varying degrees. Previous research has shown that Tai Chi can help reduce fatigue in individuals with CFS. However, the relationship between alleviating CFS-related fatigue through Tai Chi and its impact on cognitive functions remains unclear. The effects of Tai Chi on cognitive functions in individuals with CFS have not been clinically validated, and its efficacy and safety have yet to be examined through large-scale randomized controlled trials. Therefore, this protocol outlines a pilot randomized, parallel, single-blind clinical trial designed to evaluate the impact of Tai Chi therapy on fatigue and cognitive functions in individuals with CFS, using both subjective and objective assessments.

**Objective:**

This pilot study aims to explore the preliminary efficacy and safety of Tai Chi in reducing fatigue and improving cognitive function in patients with CFS, and to generate data to inform future large-scale trials.

**Methods:**

We will conduct a randomized, analyst-blinded, parallel-controlled trial with a 12-week intervention period and a 4-week follow-up. Enrolled patients will be randomly assigned to either the Tai Chi group (30 patients) or the health education group (30 patients). The Tai Chi group will receive the 24-style simplified Tai Chi intervention, while the control group will receive a health education intervention. Following the 12-week intervention, a 4-week follow-up will be conducted. The Tai Chi group will train 3 times per week, consisting of 2 in-person sessions at the Physical Education Center of Shanghai University of Traditional Chinese Medicine and 1 self-directed session guided online by an instructor. The primary outcome measure is the 20-item Multi-Dimensional Fatigue Inventory (MFI-20). The secondary outcome measures include the Montreal Cognitive Assessment (MoCA), Pittsburgh Sleep Quality Index (PSQI), Attention Network Test (ANT), working memory performance (N-Back task), and magnetic resonance imaging.

**Results:**

The research protocol and informed consent form were approved by the Shanghai Clinical Research Ethics Committee on March 18, 2024 (approval number SECCR2024-22-01). Participant recruitment began in April 2024. All interventions and concurrent data collection will be completed by October 2025, and the 4-week postintervention follow-up assessments will be finalized by the end of October 2025. Data management is still ongoing; therefore, data analysis has not yet been performed.

**Conclusions:**

As a pilot trial, the findings of this study will provide preliminary clinical evidence on the role of Tai Chi in improving cognitive function in patients with CFS and will serve as a foundation for designing future large-scale trials.

**Trial Registration:**

China Clinical Trials Registry ChiCTR2400082268; https://tinyurl.com/2tkr7j7x

**International Registered Report Identifier (IRRID):**

DERR1-10.2196/65958

## Introduction

Chronic fatigue syndrome (CFS), also known as myalgic encephalomyelitis, is a complex chronic disease characterized by pathological fatigue, generalized weakness, immune dysfunction, poor sleep, pain, and cognitive impairments [1]. Its defining feature is persistent or recurrent debilitating fatigue that is not alleviated by rest and occurs despite the absence of significant abnormalities on physical examination or laboratory tests. This condition can severely affect the work and daily life of affected individuals. The etiology and pathogenesis of CFS remain unclear; however, it is generally associated with viral infections, stress, and immune, psychological, social, and genetic factors. Furthermore, it is considered part of a group of systemic diseases caused by multisystem dysregulation [2]. In recent years, with rapid societal development, the accelerating pace of life, and increasing life pressures, many people have been in a prolonged state of excessive physical and mental fatigue. Therefore, CFS is a disease with distinct contemporary characteristics. It is prevalent among individuals in a subhealthy state across various sectors, particularly those with higher education levels, those primarily engaged in mental work, and those under high psychological stress—such as office, technology, and medical professionals. The global prevalence rate of CFS is 0.8%-3.3% [3], with a rising trend annually [4], especially among middle-aged and young populations [5]. The incidence of chronic fatigue and cognitive symptoms is substantial. Long-term, recurrent fatigue can significantly reduce quality of life and may even lead to functional disability. Patients with CFS are often forced to reduce their daily activities by as much as 50%. Furthermore, 87%-95% of patients experience nonrestorative sleep and related daytime dysfunctions [6]. Thus, CFS is considered one of the most serious public health challenges globally.

CFS can be categorized as a psychosomatic disease, primarily characterized by physical and mental fatigue, with the latter being more pronounced. It often manifests as difficulties in concentration and memory. These symptoms affect cognitive functions—such as attention and memory—to varying degrees. Cognitive impairment is the most common symptom among patients with CFS and has the greatest impact on daily functioning and learning abilities [7]. In 2021, the UK’s National Institute for Health and Clinical Excellence (NICE) included cognitive impairment (brain fog) as 1 of the 4 core symptoms of CFS. Several meta-analyses conducted in other countries have shown that patients with CFS commonly experience cognitive impairment, including dysfunctions in attention, memory, and information processing [8-10]. Approximately 89% of patients with CFS present with cognitive impairments, such as memory decline and difficulty concentrating [11]. Yongjie and colleagues [12] found that among the comorbidities of CFS, memory decline and attention deficits were the most prominent, affecting over 90% of cases. Patients with CFS exhibit impairments in immediate memory, delayed memory, and attention, with attention impairment being the most severe [13]. Some scholars believe that cognitive impairment is the most severe symptom in patients with CFS. Therefore, clinicians should remain vigilant for the development of cognitive symptoms and intervene promptly. The mechanism underlying cognitive functional impairments in patients with CFS is extremely complex and not yet fully understood. Increasing evidence suggests that disruptions to normal brain function may underlie the core symptoms of CFS, including fatigue, pain, sleep disturbances, and cognitive issues [14,15].

Currently, the etiology and mechanisms of CFS remain unclear. Diagnostic criteria are still not standardized, and highly reliable treatment methods are lacking. There are no specific diagnostic or therapeutic approaches for CFS worldwide, and most treatments remain symptomatic. Modern medicine primarily focuses on symptom management through approaches such as antianxiety therapy, immune regulation, and nutritional supplementation—aimed at alleviation rather than cure. However, the efficacy of these treatments remains controversial [16]. Moreover, they are often costly and may lead to adverse reactions such as gastrointestinal dysfunction, as well as liver and kidney damage. Patients undergoing these therapies are also at risk of relapse after discontinuing treatment. Therefore, there is an urgent need for safe and effective treatment options for CFS. In recent years, exercise therapy has emerged as a research hot spot in CFS treatment, showing notable benefits in symptom improvement. Traditional Qigong, in particular, offers several advantages, including the regulation of the body, breath, and mind. Previous studies [17,18] have shown that Qigong can relieve both overall fatigue and psychological fatigue, making it an effective treatment for CFS. As a mind-body exercise with traditional Chinese cultural roots, Tai Chi holds a unique position in alternative and traditional medicine. It engages a wide range of muscle groups, including those in the legs, trunk, and arms, and requires high levels of attention and memory. Clinical studies have shown that, compared with general aerobic exercises, Tai Chi can improve or delay the decline of overall cognitive functions, including memory, attention, and executive functions, in healthy older individuals [19,20]. It has also shown benefits in those with mild cognitive impairment [19,20], as well as in individuals with Parkinson disease [21], Alzheimer disease [22], multiple sclerosis [23], and cardiovascular diseases [24]. However, the impact of Tai Chi on cognitive functions in CFS has not yet been clinically validated, and its efficacy and safety have not been assessed in large-scale randomized controlled trials.

Regular Tai Chi practice is beneficial for relieving fatigue and improving cognition in CFS, and it is more effective than general aerobic exercise. To assess the clinical effects of Tai Chi on fatigue relief and cognitive function improvement, this study aims to design a randomized, parallel, single-blind clinical trial. The purpose of this study is to examine the effects of Tai Chi therapy on fatigue and cognitive function in CFS through both subjective and objective assessments, thereby exploring the potential links between fatigue, cognitive function, and the treatment process in CFS. This trial is currently underway, and the data will be shared through scientific publications.

## Methods

### Research Design

**Figure 1:**
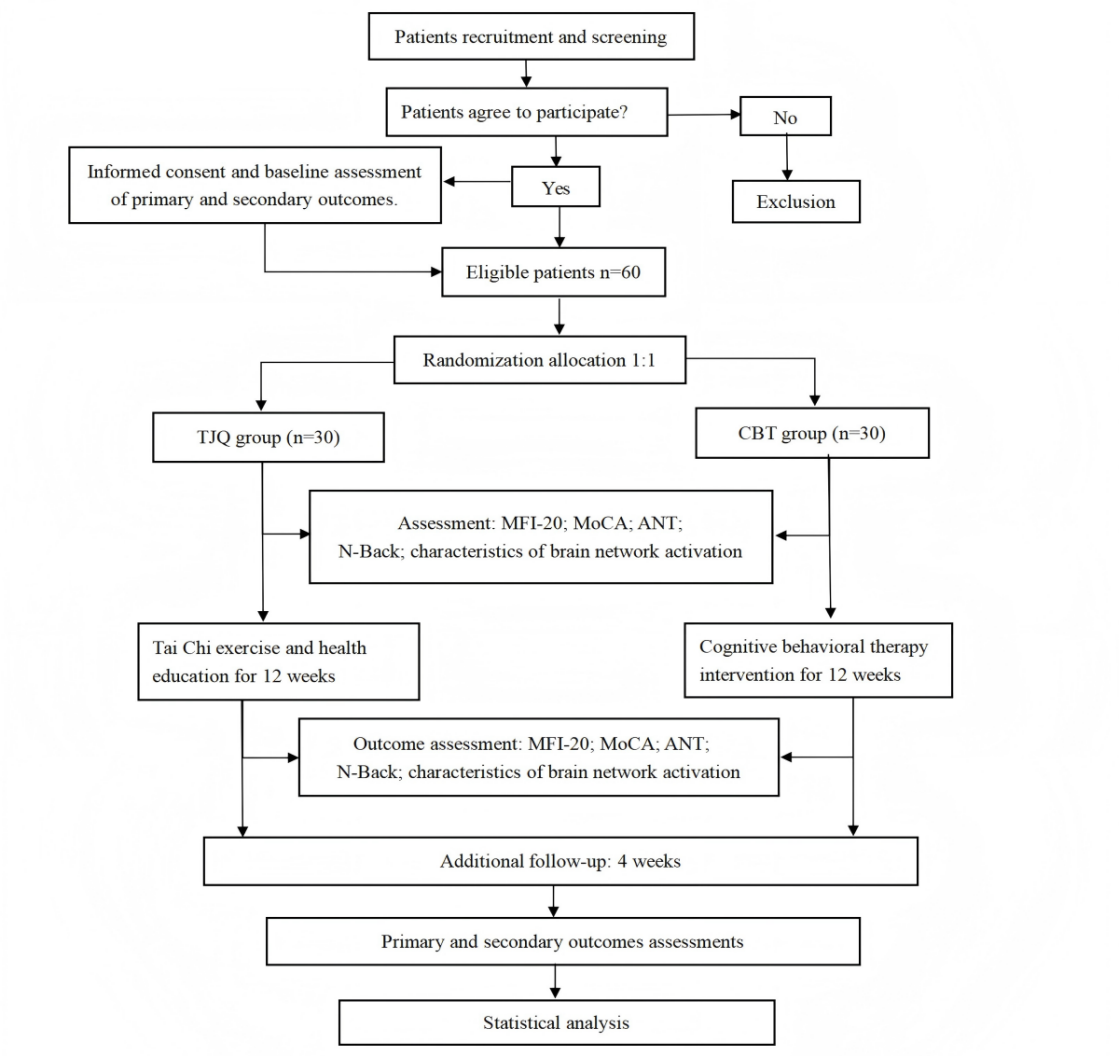
Flow diagram of the study design. ANT: Attention Network Test; CBT: cognitive behavioral therapy; MFI-20: 20-item Multi-Dimensional Fatigue Inventory; MoCA: Montreal Cognitive Assessment; TJQ: Tai Ji Quan (Tai Chi).

We designed a randomized, evaluator- and statistician-blinded, parallel-controlled trial to be conducted at Shanghai University of Traditional Chinese Medicine and The Affiliated Yueyang Hospital of Integrated Traditional Chinese and Western Medicine. Participants will be randomly allocated to either the Tai Chi group or the health education group. The Tai Chi group will receive Tai Chi intervention, while the health education group will receive cognitive behavioral therapy (CBT) intervention. The total study duration is 17 weeks: a 1-week screening period, a 12-week treatment period, and a 4-week follow-up period. The protocol version issue date is March 13, 2024. Figure 1 shows the study flowchart (see Multimedia Appendix 1 for the SPIRIT [Standard Protocol Items: Recommendations for Interventional Trials] checklist).

### Sample Size Calculation

According to Cohen [25], the ideal statistical test power and effect size should be greater than 0.8. The GPower 3.1 software (Heinrich-Heine-University) was used to conduct a 2-tailed, independent samples *t* test (paired) to calculate the sample size. Based on a previous study on CFS, the mean 20-item Multi-Dimensional Fatigue Inventory (MFI-20) score of the experimental group was 4.80 (SD 2.11), and the mean MFI-20 score of the control group was 7.13 (SD 3.29) [26]. These values were input into the GPower software to calculate the effect size (*d*=0.843, α=.05, and 1 – β=0.80), which determined the sample size for each group to be 24, accounting for a 20% dropout rate. The research group ultimately determined that the sample size for each group would be 30 participants, with a total of 60 participants. They were then divided into the Tai Chi and control groups. The sample size for this experiment provides sufficient statistical power for testing.

### Participants and Recruitment Strategy

Patients with CFS will be recruited from Shanghai University of Traditional Chinese Medicine, Yueyang Hospital of Integrated Traditional Chinese and Western Medicine, and other medical institutions. Recruitment will primarily be conducted through advertisements in the outpatient lobby, WeChat (Tencent Holdings Ltd.), and posters both inside and outside the university. All eligible participants will undergo testing at Yueyang Hospital of Integrated Traditional Chinese and Western Medicine and receive a clinical diagnosis from the physicians responsible for recruitment. Patients who meet the inclusion criteria will be selected. Before recruitment, they will be informed of the potential benefits and risks associated with participating in the exercise component of the study. Participants will then be asked to fill out an informed consent form and will be free to withdraw from the study at any time without consequence. After the intervention, patients will be informed of the study results. The results will be disseminated through peer-reviewed publications and academic conferences.

### CFS Diagnostic Criteria

The clinical diagnosis is based on the CFS diagnostic criteria published by the UK’s NICE in 2021 [27]. The criteria are as follows: (1) debilitating fatigue exacerbated by activity, not by excessive cognitive, physical, emotional, or social exertion, and not significantly alleviated by rest; (2) cognitive impairments (brain fog), including slow responsiveness, short-term memory issues, and difficulties with concentration or multitasking; (3) Postexertional malaise, with symptoms typically delayed for hours or days after activity and disproportionate to the activity, with extended recovery time, lasting for hours, days, weeks, or longer; and (4) unrefreshing sleep or sleep disturbances (or both), characterized by exhaustion upon waking, feelings of stiffness and flu-like symptoms, interrupted or shallow sleep, altered sleep patterns, or hypersomnia. All 4 symptoms must be present simultaneously and persist for at least 3 months, with other possible medical causes of fatigue ruled out. The inclusion, exclusion, and withdrawal, drop-out, and termination criteria are listed in Textbox 1.

**The inclusion, exclusion, withdrawal, drop-out, and termination criteria.**

**1. Inclusion criteria**

- Patients who meet the diagnostic criteria.
- Male or female patients aged 18-60 years and right handed.
- Patients with normal results from routine blood and urine tests, and normal liver and kidney function.
- Patients who have not received other treatments in the past month.
- Patients who agreed to participate in the study and provided informed consent.

**2. Exclusion criteria**

- Individuals who do not meet the diagnostic and inclusion criteria.
- Individuals with serious cardiovascular or psychiatric conditions.
- Individuals taking medications that may affect the outcome assessment.
- Individuals unwilling to participate in the study.
- Individuals without a primary complaint of fatigue.
- Individuals whose fatigue symptoms can be alleviated by rest.
- Individuals whose fatigue symptoms do not cause a substantial decrease in their ability to work, receive education, engage in social or recreational activities, or manage personal life responsibilities.
- Individuals with a clear diagnosis of gastrointestinal organic lesions, abnormal liver or kidney function, tumors, or other diseases.
- Pregnant or lactating women.

**3. Withdrawal, drop-out, and termination criteria**

- Individuals who do not meet the inclusion criteria and were mistakenly included.
- Participants who do not adhere to the protocol for safety evaluations.
- Participants who experience severe adverse events or complications, making it inappropriate for them to continue with the trial.

### Randomization and Allocation Concealment

Patients diagnosed with CFS who have signed the informed consent forms will be randomly assigned to either the Tai Chi group or the health education group at a 1:1 ratio. The random number table will be generated by researchers who are not involved in recruitment or intervention, using a computer. Each random number will be sealed in a pre-prepared opaque envelope with the patient’s sequence number. After providing written informed consent, participants will open an envelope labeled with their sequence number to learn which group they have been assigned to.

### Blinding

Given the nature of the interventions, the Tai Chi and health education groups cannot be blinded. However, statisticians, managers, data collectors, and outcome assessors will remain blinded during the study. Blinding will be lifted after statistical analysis is completed.

### Intervention

#### Tai Chi Group

The Tai Chi group will practice the 24-form Tai Chi, led by an instructor from Shanghai University of Traditional Chinese Medicine with at least 5 years of teaching experience. Throughout the intervention period, there will be 3 sessions per week: 2 offline classes at the Shanghai University of Traditional Chinese Medicine Sports Center and 1 self-practice session, guided online by the instructor. Each practice session lasts 60 minutes, beginning with 5 minutes of Tai Chi preexercise stretching, followed by 10 minutes of Tai Chi standing postures. The instructor will then introduce and demonstrate each movement, explain precautions, answer participants’ questions, and provide individual guidance, correcting their movements for the next 30 minutes. The session ends with 5 minutes of relaxation. For home self-practice sessions, the duration is 60 minutes, supervised via video in a WeChat group. After each practice session, a practice log will be provided, and participants will be required to fill it out. The entire treatment process lasts for 12 weeks.

#### Health Education Group

Before treatment, participants will receive information related to CFS and CBT to help them understand the principles and role of cognitive therapy in CFS treatment. The health education group will receive daily health education via a WeChat group or WeChat public account. Experts in the field will be invited to give a weekly 1-hour lecture or psychological counseling session on CFS prevention and treatment. If participants are unable to attend, lecture videos will be distributed to those who missed the session. After each learning session, a practice log will be provided, and participants will be required to fill it out. The entire treatment process lasts for 12 weeks. CBT is a psychological treatment method that combines behavioral therapy and cognitive therapy.

#### Comparator Justification

CBT was chosen as the control condition in this study based on its established role as a standard evidence-based treatment for CFS. According to the NICE guideline NG206 [27], CBT is recommended as one of the management approaches for individuals with myalgic encephalomyelitis/CFS, particularly to help them cope with the impact of symptoms and improve function. Moreover, CBT has been widely used as a comparator intervention in previous randomized controlled trials evaluating alternative or complementary treatments for CFS. Notable examples include the PACE trial, which compared CBT, graded exercise therapy, and adaptive pacing therapy, as well as other randomized controlled trials that assessed the relative effectiveness of novel interventions against CBT [28,29]. Therefore, selecting CBT as the control group in this study is methodologically appropriate and allows for a direct comparison with an established therapeutic modality.

#### Accompanying Interventions

During the study period, other CFS treatments, including both pharmacological and nonpharmacological interventions, will be prohibited. However, participants may receive treatments unrelated to CFS. Any changes in concurrent treatments will be recorded.

### Follow-Up Period

Follow-up will be conducted 4 weeks after the end of the trial, during which all participants will return to their original lifestyle. However, participants will be required to log their daily exercise or study activities. They will use WeChat to take weekly photos, providing researchers with evidence of their daily exercises or studies. At the end of the follow-up period, participants’ fatigue, attention, and working memory will be reevaluated. The follow-up assessment aims to assess the long-term effects of Tai Chi on patients with CFS. Additionally, at the end of the follow-up assessment, participants will receive a special Tai Chi souvenir to encourage their enthusiasm for the follow-up.

### Functional Magnetic Resonance Imaging Examination Procedure

Resting-state functional magnetic resonance imaging (fMRI) data will be collected using a 3.0-T Siemens magnetic resonance scanner at Yueyang Hospital of Integrated Traditional Chinese and Western Medicine and the Affiliated Shanghai University of Traditional Chinese Medicine, utilizing a 32-channel head and neck coil. The MRI sequence parameters are as follows: blood oxygen level–dependent fMRI, repetition time=2000 ms, echo time=30 ms, matrix=64 × 64, field of view=230 × 230 mm^2^, slice thickness=4 mm, gap=0.8 mm, and voxel size=3 × 3 × 3 mm^3^. Participants will arrive 30 minutes before the MRI examination and will be instructed to close their eyes and rest for 10 minutes before the scan. Throughout the experiment, participants will lie in the supine position on the examination bed and will be instructed not to move their heads during data collection. To prevent head movement, the head will be comfortably fixed with sponge pads and a head holder. Cotton ball earplugs will be provided to reduce machine noise and prevent adverse emotions. A skilled MRI technician will be assigned to complete the magnetic resonance data sampling for the participants.

### Preliminary Processing of fMRI Data

Imaging data will be processed and analyzed using MATLAB 2015a (MathWorks), SPM12 (Wellcome Department of Cognitive Neurology, UK, University College London), and VBM 8 (Voxel-Based Morphometry 8; Structural Brain Mapping Group). The raw data will be converted from DICOM to the NIFTI format, excluding the first 10 abnormal time points. MRICON will be used to convert the original scans from DICOM to the NIFTI format. The first 10 time points and images will be removed to eliminate differences in fMRI signal slice timing, adjusting the scanning time to a consistent moment. Head motion correction will be applied to remove artifacts caused by minor movements, and participants with head motion >2.5 mm (framewise displacement standard) will be excluded. The position of the anterior commissure will be manually corrected. DPABI (Data Processing & Analysis for [Resting-State] Brain Imaging) software will be used to segment 3D high-resolution structural images into gray matter, white matter, and cerebrospinal fluid, and these will be matched with the corresponding regions in the standard brain. After head motion correction, the functional images will be matched with the segmented structural images to align individual images with the standard brain template space. These images will then be further normalized to the Montreal Neurological Institute space and resampled to a voxel size of 3 × 3 × 3 mm^3^. The normalized functional images will undergo spatial smoothing with a Gaussian kernel of 4 × 4 × 4 mm^3^ full-width at half-maximum and linear detrending. High-frequency physiological noise and low-frequency drifts will be removed using a filter bandwidth of 0.01-0.1 Hz.

The amplitude of low-frequency fluctuation (ALFF) uses the amplitude of BOLD signals and the fast Fourier transform algorithm to convert the smoothed signal of each voxel from the time domain to the frequency domain. This transformation allows for the observation of changes in brain activity within the power spectrum. ALFF primarily calculates the squared values in the power spectrum within the 0.01-0.08 Hz range, reflecting the intensity of neuronal activity in various brain regions.

Functional connectivity (FC) refers to the degree of correlation between BOLD signal time series in different brain areas over time. Each voxel in a brain region contains a time series that represents the level of signal change associated with that region over time. Brain areas with significant differences in ALFF are selected as regions of interest for whole-brain FC analysis to assess changes in brain network connectivity. Changes in the time series can identify brain areas that are functionally coordinated and positively correlated with the BOLD signal, while areas negatively correlated are considered antagonistic. The most common form of FC is voxel based (seed-based FC). This process involves selecting a seed point and calculating the correlation between the BOLD signal of the seed point and that of all other voxels throughout the brain.

### Outcome Measure Assessment

The outcome measures assessed include mental and physical fatigue, overall cognitive condition, attention, working memory, sleep quality, and resting-state MRI scans. Primary and secondary outcomes, along with related self-assessment scale measurements such as the MFI-20, Montreal Cognitive Assessment (MoCA), Attention Network Test (ANT), and Working Memory Test (N-Back), will be assessed at baseline (week 1), at the end of the intervention (week 13), and at follow-up (week 18). Table 1 presents the detailed outcome assessment time points.

**Table 1:** Outline and timelines of the assessments.

Time points	Baseline (week 1)	Intervention period (weeks 2-13)	Postintervention (week 14)	Follow-up (week 18)
Inclusion criteria	✓	N/A^a^	N/A	N/A
Exclusion criteria	✓	N/A	N/A	N/A
Informed consent	✓	N/A	N/A	N/A
Randomization and allocation	✓	N/A	N/A	N/A
Intervention	N/A	✓	✓	N/A
20-item Multi-Dimensional Fatigue Inventory	✓	N/A	✓	✓
Montreal Cognitive Assessment	✓	N/A	✓	✓
Attention Network Test	✓	N/A	✓	✓
Working Memory Test (N-Back)	✓	N/A	✓	✓
Pittsburgh Sleep Quality Index	✓	N/A	✓	✓
Amplitude of low-frequency fluctuation	✓	N/A	✓	N/A
Functional connectivity	✓	N/A	✓	N/A
Adverse events	N/A	✓	✓	✓

^a^N/A: not applicable.

### Demographic and Clinical Characteristics of the Participants

The researchers will collect data on the demographic characteristics of the participants (such as age, sex, education level, marital status, living arrangements, occupation, and socioeconomic status), as well as their medical and medication history using a self-designed questionnaire. Baseline assessments will be completed before randomization.

### Primary Outcome Measure

#### MFI-20

The MFI-20 scale was developed by Smets et al [30] in 1995, after validation with patients undergoing cancer radiotherapy, individuals with CFS, psychology students, medical students, doctors, and recruits. The scale consists of 20 items, which are grouped into 5 dimensions: general fatigue, physical fatigue, reduced activity, reduced motivation, and mental fatigue. To minimize bias toward a particular direction, each dimension includes 2 statements about fatigue and 2 about the opposite (ie, not fatigued), making up a total of 4 items. Each item is scored using a 5-point Likert scale, where 1 point indicates “completely disagree” and 5 points indicate “completely agree,” resulting in a total score range of 20-100. Among these, items 2, 5, 9, 10, 13, 14, 16, 17, 18, and 19 are positively scored to reflect fatigue. Items 1, 3, 4, 6, 7, 8, 11, 12, 15, and 20 are scored inversely, reflecting the absence of fatigue. A higher score indicates more severe fatigue symptoms [31].

### Secondary Outcome Measures

#### MoCA

The MoCA scale is used to assess general cognitive status and overcomes the low sensitivity of other cognitive assessment tools to cognitive impairment. It is one of the most widely used cognitive assessment tools in clinical practice and effectively screens for cognitive dysfunction caused by various diseases, especially with high sensitivity in the early stages. It covers a wide range of cognitive domains, including orientation, attention, language, visuospatial function, memory, and executive functions. The total score is 30, with a score of 26 or higher considered normal. Scores are slightly adjusted based on educational level; for example, participants with less than 12 years of education receive an additional point added to their final MoCA score.

#### ANT

The behavioral measurement of attention is conducted using the ANT protocol proposed by Fan et al [32]. This test is administered on a computer using the E-Prime 3.0 software (Psychology Software Tools, Inc.), where stimuli are presented, and the participants’ keypress responses and reaction times for all trials are recorded. ANT measures 3 distinct attention networks: alerting, orienting, and executive control. It includes 2 main operational variables: cue type and target stimulus type. Cue types are divided into no cue, neutral cue, and spatial cue. The target stimulus types are categorized into congruent and incongruent stimuli.

The experimental stimuli consist of 3 types of original materials: the cue stimulus (“”), the target stimulus arrow (either “→” or “←”), and the fixation point (“+”). Each trial begins with a phase where the “+” fixation point appears at the center of the screen for approximately 400-1600 ms. This is followed by the cue stimulus (“”), which appears in 1 of 4 forms for approximately 100 ms. After the cue signal disappears, the fixation point (“+”) reappears alone for approximately 400 ms. Following this, the target stimulus is displayed either above or below the fixation point and remains on the screen until the participant responds. The participant’s reaction time must be less than 1700 ms. After the participant presses a key within the allotted time, the target stimulus disappears, and the fixation point (“+”) reappears in the center of the screen, signaling the start of the next trial. Each trial lasts less than 4000 ms. The cue stimulus (the asterisk “*”) can be in 1 of 4 states based on its presence and location: no cue, double cue, spatial cue, and center cue. No cue indicates that only the fixation point is displayed. The double cue indicates that asterisks appear simultaneously above and below the fixation point. The spatial cue indicates that an asterisk appears just below or above the fixation point. The center cue indicates that the asterisk is positioned in the center of the screen, replacing the fixation point. The target stimulus is a gray arrow displayed on the screen, flanked by 2 arrows on each side. These arrows may point in the same direction as the target arrow (congruent condition), in the opposite direction (incongruent condition), or be replaced by short lines (neutral condition).

In the experimental condition, participants are required to place their hands on the keyboard and focus their eyes on the fixation point in the center of the computer monitor. They must make a fast and accurate autonomous response based on the direction of the target stimulus. Specifically, if the central arrow of the target stimulus points left, participants are instructed to press the “F” key. If the central arrow points right, they should press the “J” key. The software automatically records the participant’s accuracy and reaction time. The task begins with 24 practice trials, followed by the formal experiment, which is divided into 3 blocks, each containing 96 trials. Participants are allowed to rest appropriately between blocks and can start the next block by pressing the space bar on the keyboard. The distribution of cue stimuli and target stimuli is balanced to ensure equal representation across trial types. The total duration of the task is approximately 30 minutes. The behavioral metrics include accuracy rate (higher is better), reaction time (faster is better), alerting score (no-cue reaction time [RT] to neutral-cue RT), orienting score (neutral-cue RT to spatial-cue RT), and conflict score (incongruent RT to congruent RT).

#### N-Back Test

This study uses the N-Back task paradigm to assess working memory, one of the classic experimental approaches for evaluating this cognitive function. The task is implemented using E-Prime 3.0 software and is performed on a computer with a keyboard. It presents stimuli and records participants’ reaction times and accuracy rates across all trials. During the test, participants are required to sit in front of the computer screen, maintaining a horizontal distance of 60 cm. The index fingers of the participant’s left and right hands should lightly touch the F and J keys on the keyboard, ready to respond by pressing a key when a letter appears. The N-Back paradigm includes 3 levels of memory load (0=back, 1=back, and 2=back tasks), with stimuli consisting of the letters A, B, C, D, E, and F against a black screen background with white letters. At the start of the experiment, a “+” is first displayed for 500 ms, followed by the random presentation of English letters. Each stimulus is presented for 500 ms, with an interstimulus interval of 2000 ms (blank screen). If no response is made within 2000 ms, the next stimulus is automatically presented. After each block, a prompt message saying, “Please rest for a moment; press the space bar to continue” appears. In the 0=back task, participants are instructed to compare the current letter with “X” and press the F key if they are the same or the J key if they are different. In the 1=back task, starting from the second letter, participants are instructed to determine whether the current letter is the same as the previous letter, pressing the F key if they are the same or the J key if they are different. In the 2=back task, starting from the third letter, participants are instructed to determine whether the current letter is the same as the letter 2 positions back, pressing the F key if they are the same or the J key if they are different. Participants are required to respond both quickly and accurately. The task includes 60 practice trials, followed by the formal experiment, which consists of 9 blocks, each with 30 trials (15 targets and 15 nontargets). Each memory load level includes 3 blocks, with the 0=back, 1=back, and 2=back tasks completed in sequence. Each task type involves a total of 90 stimuli (30 × 3), with a target-to-nontarget ratio of 1:1, presented in a pseudorandom order. The behavioral metrics include reaction time and accuracy rate.

In the sequence of conducting the ANT and N-Back tests, the ANT test lasts for 30 minutes, followed by the N-Back test, which lasts 15 minutes. The tests are administered consecutively, with a structured break in between. Specifically, after completing the N-Back test, participants are given a 5-minute rest period before proceeding with the ANT test. This design ensures a smooth transition while allowing participants to briefly recuperate, minimizing cognitive fatigue and maintaining optimal performance throughout the testing process. By balancing test durations and incorporating rest intervals, this approach facilitates a more reliable assessment of cognitive abilities.

#### Pittsburgh Sleep Quality Index

The Pittsburgh Sleep Quality Index (PSQI) was developed in 1989 by Buysse and colleagues [33] at the Sleep and Chronobiology Center of the Department of Psychiatry at the University of Pittsburgh Medical Center. The reliability and validity of the scale have been evaluated in China, with results showing that the index has good empirical validity. The PSQI comprises 19 items, covering 7 dimensions: subjective sleep quality, sleep duration, sleep latency (the time it takes to fall asleep), sleep disturbances, sleep efficiency, use of sleeping medication, and daytime dysfunction. The scale uses a scoring method of 0, 1, 2, and 3, with scores accumulated across dimensions, yielding a range of 0-21 points. A higher score indicates poorer sleep quality. A PSQI score of 7 is used as the cutoff point, with a score of less than 7 indicating acceptable sleep quality and more than 7 indicating poor sleep quality.

### Adverse Events and Safety

Adverse events are any unfavorable and unintended signs (including abnormal laboratory findings), symptoms, or diseases associated with the study, regardless of whether they are related to the medication used. Several studies have shown that Tai Chi is a relatively safe method for treating various diseases, including hypertension, with no reports of serious adverse reactions. If unexpected adverse events occur, defined as any functional impairments caused by the intervention, such as headaches, dizziness or vertigo, head fullness, tinnitus, chest tightness, worsening shortness of breath, increased heartbeat or palpitations, muscle soreness or pain, excessive sweating, irritability, neurasthenia, hallucinations, paranoia, or psychological stress—whether or not related to the treatment—the nearest doctor will be promptly notified for assessment and initiation of treatment.

If a serious adverse reaction occurs, the researcher will report it to the principal investigator and the ethics committee to determine whether the participant should be withdrawn from the study and treatment. The researchers will make every effort to prevent and treat any harm that may arise from this study. If the expert committee believes that the adverse event is related to the Tai Chi treatment, the research team will cover the treatment costs and provide appropriate financial compensation for damages related to the trial.

### Quality Control

The project leader will be responsible for the design, coordination, and quality control of the entire study. All researchers will undergo uniform training before the data collection period. Before data collection, patients are required to maintain a healthy lifestyle, such as avoiding staying up late and abstaining from alcohol, to prevent the influence of unstable factors. During the study, all data will be statistically analyzed by trained independent assessors. We will collect outcome data from all participants, regardless of whether they complete the study. The Data and Safety Monitoring Board will consist of an internal medicine physician, a Tai Chi instructor, a medical statistician, an ethicist, and a radiologist, and will conduct assessments throughout the study.

To ensure that the patients in the Tai Chi group can practice correctly at home, we will provide them with a video of Tai Chi practice methods recorded by a national Tai Chi champion, along with a booklet containing specific practice details. The Tai Chi group will also receive a practice record that will be filled out after each practice session.

### Data Collection and Management

Data entry and database creation will be handled by 2 data managers who are not part of the research team and are unaware of the group assignments. Everyone participating in the trial will have access to the full data set. All original data will be retained for clinical data analysis and safety evaluation. All research-related original data will be stored at Shanghai University of Traditional Chinese Medicine.

### Informed Consent

Before the trial begins, participants will be informed about the research process, their responsibilities, and the physical examinations and precautions involved. Most importantly, they will be informed that their participation is entirely voluntary and that they can refuse or withdraw at any time without affecting their medical care or other benefits. If a participant withdraws, the collected data will not be deleted and will be included in the final analysis. A written informed consent form will be obtained from each participant before starting any treatment related to the study. The research assistants will be responsible for obtaining informed consent from all participants.

### Statistical Analysis

All statistical analyses and processing will be independently performed by a researcher who did not participate in the treatment or scale assessments during the process. The Statistical Package for the Social Sciences version 20.0 (IBM Corporation) will be used for statistical analysis. Quantitative data with a normal distribution will be described as mean (SD). For quantitative data with a nonnormal distribution and outcome indicators, the rank-sum test will be used. A *P* value of <.05 indicates a statistically significant difference.

For the neuropsychological test outcome indicators, ANT, and N-Back test behavioral metrics, a 2 (group: Tai Chi group, education group) × 2 (time: pretest, posttest) repeated measures ANOVA will be used. For cases not meeting the sphericity test, the Greenhouse-Geisser method will be applied to correct the statistical data (degrees of freedom and *P* value), and Bonferroni correction will be applied for post hoc comparisons. All statistical inferences will be 2-tailed, with the level of significance set at α=.05. A *P* value of <.05 indicates statistically significant differences.

### Statistical Analysis of Neuroimaging Results

The ALFF values and FC between the Tai Chi group and the health education group will be compared using independent samples *t* tests. A 2-sample *t* test will be used to compare the ALFF values between the 2 groups. Pre- and postintervention data within groups will be compared using paired *t* tests. After the ALFF analysis, the maximum peak coordinates of different brain regions will be selected, and the cluster at the peak coordinates will be considered the region of interest. Differences in FC of the region of interest with the whole brain will be calculated. Correlation analyses will be conducted using Pearson correlation coefficients based on clinical and fMRI imaging data. Missing data will be processed using the multiple imputation method.

### Ethical Considerations

This study follows the Declaration of Helsinki (as revised in 2013) and the International Ethical Guidelines for Biomedical Research Involving Human Subjects (Articles 37 and 38). The study protocol and consent forms have been approved by the Shanghai Clinical Research Ethics Committee (approval number SECCR2024-22-01). They were registered on March 26, 2024, in the Clinical Trial Registry (a World Health Organization International Clinical Trials Registry Platform [WHO ICTRP] member) under registration number ChiCTR2400082268.

All participants will be fully informed of the inclusion and exclusion criteria for this study before the trial begins. They will sign informed consent forms before the trial and be informed that they have the right to withdraw from the trial at any time. Recruitment and randomization will be conducted transparently. If changes occur in our research protocol, a written application will be submitted to the research ethics committee. The committee members will decide whether it is necessary to modify the protocol. After the clinical study is completed, a study completion report will be submitted and published in academic journals.

## Results

Participant recruitment commenced in April 2024. All interventions and concurrent data collection will be completed by October 2025. The 4-week postintervention follow-up assessments will be finalized by the end of October 2025. Data management is currently ongoing, and statistical analysis has not yet been performed.

## Discussion

To the best of our knowledge, this study is the first to explore the effects of Tai Chi on CFS from a cognitive functional perspective. To validate the specific role of Tai Chi in CFS treatment, this study aims to evaluate the impact of Tai Chi on fatigue, cognitive functions, and changes in brain function using different clinical scales, psychological experimental paradigms, and neuroimaging scans. Currently, it is widely accepted in the academic community that CFS can cause cognitive impairments. However, some reports occasionally yield inconsistent results [34,35], possibly because early mild cognitive impairments may not be fully detected by conventional neuropsychological scales and can easily be overshadowed by other clinical symptoms. In most studies, subitems measuring cognitive functions, such as attention and memory, are typically included in the composite scores of scales for other cognitive domains or in overall cognitive assessment paradigms. Moreover, due to the strong subjectivity of scale assessments and the low sensitivity of indicators, there are certain limitations. With the use of various cognitive tasks and different methods of assessing cognitive functions, direct comparisons are not feasible, leaving a lack of more direct evidence for assessing cognitive functions in patients with CFS. Therefore, there is an urgent need to apply more precise and scientific neuropsychological experimental paradigms to assess specific cognitive domains, providing objective and accurate data to support the assessment of cognitive impairments in CFS.

Togo [36] showed that the ANT has clinical value for assessing the cognitive functions of patients with CFS. Some scholars have proposed using continuous attention assessment as a reliable biomarker for diagnosing CFS [37]. Nevertheless, there is still a lack of comprehensive ANT paradigm evaluations and analyses of attention network functions in patients with CFS. The N-Back test is a commonly used paradigm in cognitive psychology for assessing working memory capacity [38], and the N-Back task paradigm, a continuous processing operation task, is one of the most widely used experimental paradigms in working memory brain imaging studies.

This study uses more precise and scientific neuropsychological experimental paradigms, such as the ANT and N-Back, to assess specific domains of cognitive impairments in CFS. It provides objective and accurate data to support the assessment of cognitive impairments in CFS. This approach offers a theoretical basis for the early clinical diagnosis and intervention of cognitive function impairments in patients with CFS, offering significant guidance.

Resting-state fMRI studies are essential for elucidating the intrinsic functional organization of the brain and can enhance our understanding of brain functional changes in CFS and its treatment. Neuroimaging scans can reveal changes in brain activation and FC in patients with CFS after Tai Chi interventions, providing clear, effective, and direct evidence. Previous fMRI studies have found brain functional changes or metabolic abnormalities in areas such as the prefrontal cortex, posterior cingulate cortex, parieto-occipital cortex, hippocampus, parahippocampal gyrus, and precuneus in patients with CFS. These changes in the central nervous system may serve as a pathological basis for the physical fatigue and cognitive impairments observed in patients with CFS [39,40].

The academic community has conducted clinical research on mind-body exercise interventions for CFS from various perspectives, yielding a wealth of effective research outcomes. Tai Chi emphasizes the unity of form and spirit, practiced with the guidance of mind over energy. It stresses the coordination of body and mind, advocating stillness and emphasizing nourishment. It can improve insomnia and fatigue symptoms in patients with CFS without toxic side effects. A meta-analysis showed that Tai Chi can improve fatigue more effectively than traditional therapies and has a positive effect on cancer-related fatigue [41]. Kuangshi et al [42] recruited 21 patients with CFS, with 19 in the control group, implementing a 4-week 24-form Tai Chi training and conducting functional brain MRI. Results showed that, before practicing Tai Chi, the experimental group exhibited significantly weakened FC in the default mode network, compared with the control group, in regions including the left superior temporal gyrus and the left dorsolateral prefrontal cortex. Meanwhile, significantly enhanced FC was observed in the right lateral temporal lobe. Therefore, Tai Chi has a characteristic modulatory effect on abnormal activity in the default mode network of patients with CFS .

Kuangshi et al [43] showed that, after practicing Tai Chi, the experimental group exhibited significantly reduced functional ALFF values in brain regions including the left orbitofrontal/cortical pole, right anterior cingulate gyrus, and right lateral temporal lobe. However, significantly increased functional ALFF values were observed in the left sensorimotor cortex and the right visual cortex. Therefore, Tai Chi exercise can improve fatigue and sleep in patients with CFS, enhancing their quality of life, possibly due to its specific effect on the brain’s sensorimotor cortex, left frontal lobe, right lateral temporal lobe, anterior cingulate gyrus, and visual cortex. This research provides biomarkers for subsequent studies and imaging support for the central mechanism of Tai Chi. However, as an exploratory study with a small sample size, it does not include groups practicing aerobic exercise or other interventions, which would help determine the specificity of the Tai Chi intervention.

Wu et al [44] designed a longitudinal trial with Tai Chi as the intervention therapy, using the FCs of the whole brain as exploratory variables to detect abnormalities in the brain’s connectivity structure in patients with CFS and explore changes during Tai Chi exercise. Our study explored the impact of Tai Chi on brain FC in CFS. Results showed that Tai Chi could improve fatigue, sleep quality, and physical health status in patients with CFS by enhancing the FCs within the left frontoparietal network and default mode network [42].

The results of this trial can evaluate the efficacy of Tai Chi in treating fatigue symptoms and cognitive impairments in patients with CFS by assessing changes in brain activation, ANT and N-Back results, and related clinical scale outcomes.

Despite the promising hypothesis of this study, it also has some notable limitations. First, the screening for inclusion criteria is solely based on self-assessment scales without the use of specialized psychometric testing equipment, which may lead to less stringent inclusion of patients with CFS. However, by limiting participants’ ages to 18-60 years, the likelihood of chronic fatigue caused by medical and psychiatric conditions is reduced. Thus, the study results cannot be generalized to other populations. Second, in this study, the intervention is a nonpharmacological treatment, which cannot be ideally implemented according to double-blind standards. Ideally, each participant should be blinded to the trial; however, this is challenging to achieve in nonpharmacological trials. Hence, bias may be inevitable. However, to ensure the authenticity of the experimental data and reduce biases during the experiment, we will rigorously randomize participants and ensure that the interveners, assessors, and statisticians remain blind to each other. Additionally, the neuroimaging study in this trial is only at an exploratory stage with a limited sample size. In future research, increasing the sample size could provide more persuasive evidence. Finally, this trial only conducted a 4-week follow-up. Therefore, the follow-up period should be extended to validate the long-term clinical outcomes and potential effects of Tai Chi.

Furthermore, this study was designed as a pilot randomized controlled trial, with the primary aim of assessing the feasibility and preliminary efficacy of Tai Chi therapy in patients with CFS. The relatively small sample size limits the statistical power and generalizability of the results; therefore, all findings should be interpreted with caution. The outcomes of this pilot study will help inform the design and methodology of future large-scale, multicenter trials.

Despite the limitations mentioned, this study still possesses several advantages. Compared with other treatment methods, such as medication and psychological counseling, Tai Chi offers significant benefits for patients with CFS. It is easy to learn, not restricted by time or space, and can be practiced independently for therapeutic purposes. Moreover, this is the first study to investigate Tai Chi intervention for cognitive impairments in CFS. More importantly, it will objectively demonstrate the efficacy of Tai Chi in addressing cognitive impairments in CFS using precise, sensitive computerized neuropsychological experimental paradigms and high-spatial-resolution fMRI technology. Finally, the use of posters and social platforms for recruitment aims to include individuals from various social strata, thereby enhancing the representativeness of the sample.

In conclusion, this protocol outlines a rigorous, well-structured pilot randomized controlled trial designed to evaluate the preliminary efficacy and safety of Tai Chi for cognitive impairments in CFS.

While the findings may not be definitive, they will provide important initial evidence and contribute to the foundation for future larger-scale studies.

## Appendix

Multimedia Appendix 1 SPIRIT checklist.

## Abbreviations

ALFF: amplitude of low-frequency fluctuation
ANT: Attention Network Test
CBT: cognitive behavioral therapy
CFS: chronic fatigue syndrome
DPABI: Data Processing & Analysis for (Resting-State) Brain Imaging
FC: functional connectivity
fMRI: functional magnetic resonance imaging
MFI-20: 20-item Multi-Dimensional Fatigue Inventory
MoCA: Montreal Cognitive Assessment
MRI: magnetic resonance imaging
NICE: National Institute for Health and Clinical Excellence
PSQI: Pittsburgh Sleep Quality Index
RT: reaction time
SPIRIT: Standard Protocol Items: Recommendations for Interventional Trials
WHO ICTRP: World Health Organization International Clinical Trials Registry Platform
